# Simultaneous Extraction of the Grain Size, Single-Crystalline Grain Sheet Resistance, and Grain Boundary Resistivity of Polycrystalline Monolayer Graphene

**DOI:** 10.3390/nano12020206

**Published:** 2022-01-09

**Authors:** Honghwi Park, Junyeong Lee, Chang-Ju Lee, Jaewoon Kang, Jiyeong Yun, Hyowoong Noh, Minsu Park, Jonghyung Lee, Youngjin Park, Jonghoo Park, Muhan Choi, Sunghwan Lee, Hongsik Park

**Affiliations:** 1 School of Electronic and Electrical Engineering, Kyungpook National University, Daegu 41566, Korea; hoepark@ee.knu.ac.kr (H.P.); jyl2015@ee.knu.ac.kr (J.L.); chjlee@knu.ac.kr (C.-J.L.); jwnkang@knu.ac.kr (J.K.); yeong112@knu.ac.kr (J.Y.); hwn1327@knu.ac.kr (H.N.); msp7352@knu.ac.kr (M.P.); jongh1019@knu.ac.kr (J.L.); dudwls1218@knu.ac.kr (Y.P.); jonghoopark@knu.ac.kr (J.P.); mhchoi@ee.knu.ac.kr (M.C.); 2 School of Electronics Engineering, Kyungpook National University, Daegu 41566, Korea; 3 School of Engineering Technology, Purdue University, West Lafayette, IN 47907, USA; sunghlee@purdue.edu

**Keywords:** CVD graphene, polycrystalline, grain size, single-crystalline grain, grain boundary (GB), GB distribution, sheet resistance, transmission-line model measurement

## Abstract

The electrical properties of polycrystalline graphene grown by chemical vapor deposition (CVD) are determined by grain-related parameters—average grain size, single-crystalline grain sheet resistance, and grain boundary (GB) resistivity. However, extracting these parameters still remains challenging because of the difficulty in observing graphene GBs and decoupling the grain sheet resistance and GB resistivity. In this work, we developed an electrical characterization method that can extract the average grain size, single-crystalline grain sheet resistance, and GB resistivity simultaneously. We observed that the material property, graphene sheet resistance, could depend on the device dimension and developed an analytical resistance model based on the cumulative distribution function of the gamma distribution, explaining the effect of the GB density and distribution in the graphene channel. We applied this model to CVD-grown monolayer graphene by characterizing transmission-line model patterns and simultaneously extracted the average grain size (~5.95 μm), single-crystalline grain sheet resistance (~321 Ω/sq), and GB resistivity (~18.16 kΩ-μm) of the CVD-graphene layer. The extracted values agreed well with those obtained from scanning electron microscopy images of ultraviolet/ozone-treated GBs and the electrical characterization of graphene devices with sub-micrometer channel lengths.

## 1. Introduction

Chemical vapor deposition (CVD) is the most effective method for uniformly growing monolayer graphene on a wafer scale in a reproducible way [1]. However, CVD graphene can typically be grown as a polycrystalline structure composed of multiple single-crystalline grains connected by disordered grain boundaries (GBs) [2,3,4,5,6]. Scattering at the GB (i.e., structural line defect) affects carrier transport, as does scattering within a single-crystalline grain; thus, both the GB and grain act as major resistive sources in polycrystalline graphene [4,5,6]. Because the electrical properties of graphene are determined by the competition between these two resistive sources—i.e., relatively high-resistive GB and low-resistive grain—the average grain size has a significant impact on the electrical properties [7,8,9,10]. Therefore, unlike single-crystalline graphene, whose electrical performance can be explained only by the sheet resistance of the layer, the performance of polycrystalline CVD graphene should be explained by a combination of various grain-related parameters, such as the average grain size, single-crystalline grains sheet resistance, and GB resistivity. For this reason, rigorous evaluation of these grain parameters is crucial for the design and fabrication of CVD-graphene devices.

Accordingly, various techniques for characterizing grain parameters have been actively studied over the last decade. In the case of grain size, it can be evaluated through structural characterization and identification of GBs using spectroscopic or microscopic measurements. For instance, spatial mapping of the Raman peak intensities for graphene (D peak at ~1350 cm^−1^, G peak at ~1580 cm^−1^, and 2D peak at ~2690 cm^−1^) enables the location and shape of GBs and grains to be identified [11]. This is an effective method for evaluating the size of individual grains, but estimating the average grain size of CVD graphene across the entire grown region is difficult because of the limited inspection area and the extremely slow mapping speed. Instead of mapping the Raman peaks, the GBs with an angstrom-scale width can be imaged directly by performing an ultraviolet (UV)/ozone treatment after growing graphene on a Cu substrate. The UV/ozone treatment selectively oxidizes Cu beneath the GBs through strong chemical reactions with O and OH radicals, allowing the GBs to be visualized and examined under an optical microscope (OM) or a scanning electron microscope (SEM) [12,13]. Although this process provides a convenient way to observe multiple grains and to evaluate their sizes, for a global estimation of the average grain size, a time-consuming manual process that evaluates the sizes of individual grains from a large amount of microscopy images covering a wide area of CVD-grown graphene should accompany it [14].

For the electrical properties of GBs and single-crystalline grains, four-terminal measurement-based evaluation techniques have been widely used. To extract the resistances of a GB and grain separately using these techniques, the location of the GB should first be identified with non-destructive transmission electron microscopy (TEM) [6]. An electron-beam lithography system is then used to fabricate a Hall-bar pattern across two grains joined by the GB. The sheet resistance of the single-crystalline grain and the resistivity of the GB can be extracted by performing a series of four-terminal measurements with this pattern [6]. This technique is significantly useful for understanding the electrical properties of individual grains and GBs; however, high-level technical skills are required to accurately estimate the location of the GB and fabricate the Hall-bar pattern aligned well with the GB location [4]. Furthermore, because of the limited TEM resolution capable of identifying the GBs, it is difficult to extend this technique to a global evaluation method that can extract the average grain sheet resistance and GB resistivity from multiple single-crystalline grains and GBs. As an alternative approach, an electrical characterization technique based on ohmic scaling law was developed for the global evaluation of these two electrical parameters [4]. In this technique, the average grain sheet resistance and GB resistivity can be extracted on a large scale by measuring the channel sheet resistance of each CVD-graphene sample as a function of the average grain size and then fitting it to the ohmic scaling law (i.e., a simple 1D series-resistance model) [4,5]. However, to apply this technique effectively, it is necessarily required to investigate the exact average grain size of each graphene sample, and further, prepare multiple CVD graphene samples with different average grain sizes but identical average grain sheet resistance and GB resistivity. These requirements may limit the practical application and an accurate extraction of the average grain sheet resistance and GB resistivity.

In this paper, we propose for the first time an electrical characterization method for extracting the average grain size, grain sheet resistance, and GB resistivity of monolayer CVD graphene simultaneously. For this purpose, we investigate the probability distribution of the number of GBs depending on the graphene–channel dimension, from which we develop an analytical resistance model that can explain the relationship between the electrical properties of polycrystalline graphene and its grain parameters. With this resistance model, we show that the three-grain parameters can be extracted simultaneously with an accuracy greater than 99% from the dependence of the material’s electrical property (i.e., sheet resistance) on the channel dimension. To validate the developed resistance model and its applicability for parameter extraction, we fabricate a transmission-line model (TLM) pattern on monolayer CVD graphene and characterize the channel sheet resistance (*R_sh_*) as a function of channel length (*L_ch_*). We show that the average grain size, grain sheet resistance, and GB resistivity of CVD graphene can be extracted simultaneously from the analytical resistance model that is fitted to the measured *R_sh_*–*L_ch_* curve. The three extracted values are then compared with those obtained using conventional methods and those reported in the literature.

## 2. Materials and Methods

### 2.1. Graphene Growth and Transfer

After the development of an analytical resistance model, to verify its applicability for grain-parameter extraction, we synthesized monolayer graphene on a 25-μm-thick Cu foil (99.8% purity, Alfa-Aesar Inc., Ward Hill, MA, USA) by a thermal CVD system (TCVD 100, Scientec Inc., Suwon, South Korea). To grow the polycrystalline graphene layer with uniform-size grains, electropolishing of the Cu surface was first performed in an 85% phosphoric acid bath using a constant voltage of 1.2 V for 20 min [15]. The polished Cu foil was then loaded into the CVD chamber and annealed at 1050 °C for 1 h with a flow of Ar (570 sccm) and H_2_ (100 sccm) for surface treatment. Following the annealing step, monolayer graphene was grown at 1050 °C for 1 h under a chamber pressure of 2 Torr with a flow of Ar (570 sccm), H_2_ (100 sccm), and CH_4_ (2 sccm). The CVD chamber was then cooled down to room temperature in an Ar environment before the as-grown graphene sample was finally removed from the chamber.

For device fabrication, CVD-grown monolayer graphene was transferred onto thermally oxidized Si substrates (90-nm-thick SiO_2_) by the optimized poly (methyl methacrylate) (PMMA)-mediated transfer method [16]. First, a PMMA solution (495 A4, MicroChem Inc., Westborough, MA, USA) was spin-coated onto the top side of the graphene-grown Cu foil at 1000 rpm for 60 s, followed by drying at room temperature for 24 h. The graphene layer grown on the back side of Cu was etched using O_2_ plasma ashing (RF power = 30 W, working pressure = 30 m Torr) for 3 min. The Cu foil was then etched using diluted ammonium persulfate solution (0.02 M, Sigma Aldrich Inc., St. Louis, MO, USA) at room temperature for 24 h, after which the remaining PMMA/graphene stack was rinsed repeatedly with deionized water. The rinsed PMMA/graphene stack was finally transferred onto the oxidized Si substrate and the transferred sample was baked at 160 °C in a vacuum for 30 min to remove residual water and improve the adhesion between the graphene layer and the substrate. To minimize the PMMA residue and wrinkles, the PMMA layer was removed using acetic acid at room temperature for 3 h, followed by annealing at 300 °C for 3 h under an ultrahigh vacuum (~10^−8^ Torr) [16].

### 2.2. Device Fabrication

To extract the grain parameters of CVD graphene using the developed analytical resistance model, TLM patterns composed of back-gated field-effect transistors (FETs) with varying channel lengths (2–100 μm) were fabricated on CVD graphene transferred onto 90-nm-thick oxidized Si substrates. First, channel regions of the FETs were patterned by *i*-line mask-aligner lithography (MA6/BA6, Karl Suss Inc., Munich, Germany) with a positive-tone photoresist (AZ GXR-601, AZ Electronic Materials Inc., Branchburg, NJ, USA), and graphene channels were defined using O_2_ plasma etching (30 W, 30 m Torr) for 2 min. Following the channel definition, the second photolithography process was performed using an image reversal photoresist (AZ 5214-E, AZ Electronic Materials Inc.) for source/drain electrode patterning. Then, a 20-nm-thick Pd and 50-nm-thick Au were sequentially deposited by an electron-beam evaporation system (SRN-200, Sorona Inc., Anseong, South Korea) for contact formation, after which the residual photoresist layer and the metal deposited on top of the photoresist were removed by the lift-off process in a warm acetone bath. CVD-graphene FETs with sub-micrometer channel lengths (0.18–0.75 μm) were fabricated using electron-beam lithography (Raith 150-TWO, Raith Inc., Dortmund, Germany) and a positive-tone electron-beam resist (AR-P 672.045, Allresist Inc., Strausberg, Germany). For patterning the graphene channels and source/drain electrodes, electron-beam conditions of an area dose of 200 μC/cm^2^, a step size of 5 nm, and an acceleration voltage of 30 kV were used.

### 2.3. Characterizations

The electrical characteristics of the fabricated graphene FETs were measured using a semiconductor parameter analyzer (4156C, Agilent Inc., Palo Alto, CA, USA) in a probe station under a high vacuum (~10^−7^ Torr). Before the measurements, the graphene surface was annealed at 120 °C for 3 h in the vacuum probe station to remove any moisture, oxygen, and photoresist residue, which act as p-type dopants [16,17,18]. A Raman spectroscope (inVia reflex, Renishaw Inc., Wotton-under-Edge, UK) with 532-nm excitation was used to evaluate the material quality of monolayer CVD graphene. The top-view images of the CVD-graphene surface and fabricated graphene FETs were obtained using a field-emission SEM (SU8220, Hitachi Inc., Tokyo, Japan) and an OM (BX51, Olympus Inc., Tokyo, Japan) system. The GB visualization for estimating the average grain size was performed in a UV/ozone chamber (PSD-Pro, Novascan Technologies Inc., Boone, IA, USA) by irradiating a 254-nm UV light with an output power of 20 mW/cm^2^ under ambient conditions. The grain sizes of CVD graphene observed in the top-view SEM images after the UV/ozone treatment were measured using the ImageJ software provided by the National Institutes of Health, USA. Note that the grain-size estimation from the SEM images was performed to check the accuracy of the average grain size extracted by the proposed electrical characterization method.

## 3. Results and Discussion

**Figure 1:**
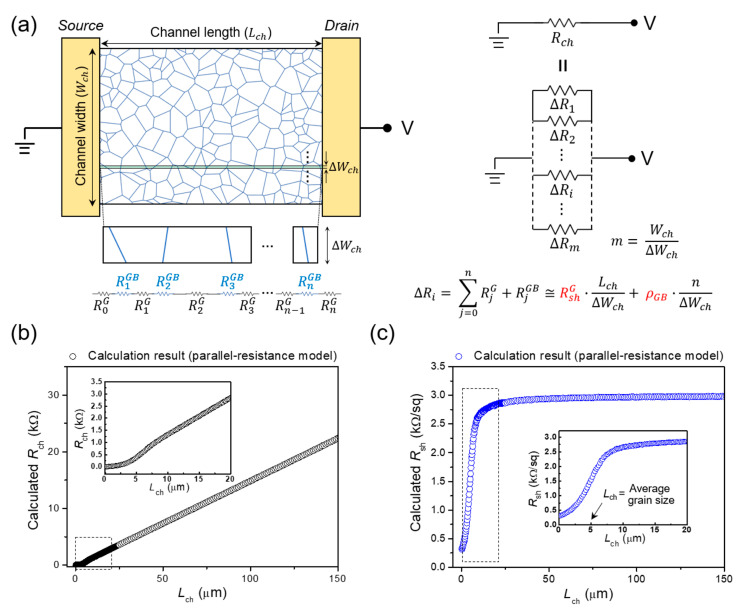
Parallel-resistance model for theoretical computation of the sheet resistance depending on the channel dimension. (**a**) A schematic of the electrical device with a poly-graphene channel simulated using the VT method. For poly-graphene resistance modeling, the channel width is divided into extremely narrow elements (∆*W_ch_* = *W_ch_*/10^5^). The resistance of the poly-graphene channel is calculated from the parallel connection of the divided elements. (**b**) The calculated channel resistance as a function of channel length for polycrystalline graphene (with an average grain size of 5 μm) simulated using the VT method. Inset: the calculated channel resistances in relatively short channels (denoted by the dashed box), which shows that the slope (d*R_ch_*/d*L_ch_*) varies with the channel length. (**c**) The calculated channel sheet resistance as a function of the channel length. Inset: the calculated channel sheet resistances in relatively short channels (denoted by the dashed box), which shows that the sheet resistance decreases significantly as the channel length approaches the average grain size.

To develop a characterization method for extracting the average grain size, grain sheet resistance, and GB resistivity, it is necessary to investigate the effects of these parameters on the electrical characteristics of polycrystalline-graphene devices. Thus, we first theoretically calculated the channel sheet resistance of polycrystalline graphene as a function of channel length using a parallel-resistance model [14], and investigated its dependence on the channel length. For the sheet-resistance computation, the Voronoi tessellation (VT) method was used to generate 2D polycrystalline structures (Figure 1a), which can depict a real polycrystalline morphology with non-uniform sizes and shapes of grains [14,19,20,21,22]. Because the sizes and shapes of polycrystalline graphene are not uniform, the number of GBs impeding carrier transport between two electrodes varies with the location in the poly-graphene channel. This indicates that the sheet resistance of the graphene channel can vary locally—i.e., the sheet resistance would not be uniform within the polycrystalline channel. Thus, for rigorous resistance modeling that takes into account the local non-uniformity of the GB number, the channel width was divided into extremely narrow elements, and the number of GBs within each width element was counted separately. The resistance of each width element, which is the sum of the resistances of numerous grains and GBs connected in series (Figure 1a), was then calculated from the following 1D ohmic scaling law [4]:

(1) $$\Delta R_{i}=R_{sh}^{G}\frac{L_{ch}}{\Delta W_{ch}}+\rho_{GB}\frac{n_{i}}{\Delta W_{ch}}$$

where ∆*R_i_* is the resistance of each width element, $R_{sh}^{G}$ is the average sheet resistance of single-crystalline grains, *ρ_GB_* is the average resistivity of GBs, *L_ch_* is the channel length, *W_ch_* is the channel width, and *n_i_* is the number of GBs within each width element. Subsequently, the total channel resistance of polycrystalline graphene was calculated from the sum of the resistances of every width element connected in parallel (Figure 1a). The equation for calculating the channel resistance as a function of the channel length is as follows:

(2) $$\frac{1}{R_{ch}\left(L_{ch}\right)}=\overset{m}{\underset{i=1}{\sum }}\frac{1}{\Delta R_{i}}=\overset{m}{\underset{i=1}{\sum }}\frac{1}{R_{sh}^{G}\frac{L_{ch}}{\Delta W_{ch}}+\rho_{GB}\frac{n_{i}}{\Delta W_{ch}}}$$

where *R_ch_* is the channel resistance and *m* is the number of divided width elements (=*W_ch_*/∆*W_ch_*). In this study, the channel width was divided into 10^5^ elements for the parallel-resistance modeling. Furthermore, to estimate the average channel resistance as a function of the channel length, we randomly generated 1000 polycrystalline structures with an average grain size of 5 μm using the VT method and repeated this calculation process (see Figure S1 for the calculation results of poly-graphene channels with different average grain sizes of 2.5 and 15 μm). The *W_ch_* used in the calculation was 20 μm, and the $R_{sh}^{G}$ and *ρ_GB_* values were 300 Ω/sq and 10.6 kΩ-μm, respectively, which were selected within the range of those of CVD graphene reported in the literature [4,5]. (The results calculated using different *W_ch_*, $R_{sh}^{G}$, and *ρ_GB_* values are shown in Figure S2). Note also that the source/drain contact resistance was not taken into account in the resistance modeling and computation processes. The theoretically calculated average resistance of the poly-graphene channel (average grain size of 5 μm) simulated using the VT method is shown in Figure 1b. The channel resistance is directly proportional to the channel length in the long-channel region (i.e., constant d*R_ch_*/d*L_ch_*), whereas the slope (d*R_ch_*/d*L_ch_*) varies with the channel length in the relatively short-channel region (particularly the *L_ch_* around the average grain size). This indicates that the sheet resistance of the polycrystalline channel depends on the channel dimension, unlike the single-crystalline graphene or other single-crystalline semiconductors. The average channel sheet resistance (*R_sh_*) as a function of channel length is shown in Figure 1c. The sheet resistance was calculated from *R_ch_* × *W_ch_*/*L_ch_*. The channel sheet resistance is constant for long channels; however, it decreases sharply as the channel length is reduced below approximately 25 μm. The decrease in channel sheet resistance is most prominent at channel lengths around the average grain size, which is due to the significantly lowered probability of the existence of GBs at those channel lengths [14]. This implies that the GB density and distribution within the channel region play a critical role in determining the dependence of the channel sheet resistance on the channel length.

**Figure 2:**
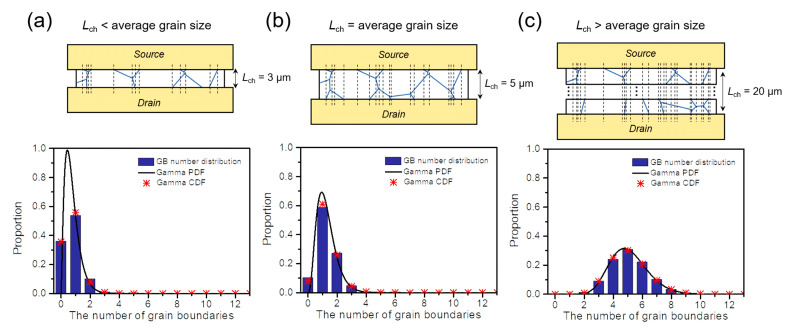
Histogram distributions of the proportion of the GB number within the channel region when the channel length is (**a**) less than the average grain size, (**b**) equal to the average grain size, and (**c**) greater than the average grain size. Each distribution was evaluated by counting the GB number within narrow width elements divided into 10^5^. The black dashed lines in (**a**–**c**) indicate the locations where the number of GBs changes. For all channel lengths, the envelope of the proportional distribution of the number of GBs follows the continuous probability density function of the gamma distribution (gamma PDF), and the discrete proportion distribution of the GB number can be estimated from the cumulative distribution function of the gamma distribution (gamma CDF) with an accuracy greater than 98%.

As shown in Figure 1, it is critical to estimate the GB density and distribution within the graphene channel to understand the dependence of the channel sheet resistance on the channel length. Thus, we investigated the proportion distribution of the number of GBs depending on the channel length by counting the GB number within narrow width elements divided into 10^5^. For statistical evaluation, we repeated the process with 1,000 polycrystalline-graphene structures (average grain size of 5 μm) generated randomly using the VT method, as in the channel resistance calculation. The average histogram distributions of the proportion of the GB number within the channel region at three different channel lengths are shown in Figure 2a–c (see Figure S3 for the results at various channel lengths). The number of GBs within the channel region is limited to 0–3 at channel lengths less than or equal to the average grain size (i.e., *L_ch_* ≤ 5 μm), whereas it is evenly distributed at the channel length greater than the average grain size. This explains why there is a significant decrease in sheet resistance at channel lengths near the average grain size (Figure 1c). We investigated several distribution functions to find a way to estimate such a proportion distribution without counting the number of GBs and found that the envelope of the proportional distribution of the GB number follows the continuous probability density function of the gamma distribution (i.e., gamma PDF) [23]:

(3) $$GamPDF\left(x\right)=\frac{1}{\beta^{\alpha }\int_{0}^{\infty }u^{\alpha -1}e^{-u}du}x^{\alpha -1}e^{-\frac{x}{\beta }}\;for\;x,\;\alpha ,\;\beta >0$$

where *α*∙*β* is the mean and *α*∙*β*^2^ is the variance. When *α* = 3.85 × *L_ch_*/*l_G_* and *β* = 0.33 (where *l_G_* is the average grain size), it was empirically confirmed that the gamma PDF agrees well with the envelope of the proportional distribution of the GB number for all channel lengths (Figure 2 and Figure S3). Furthermore, this observation was also valid for poly-graphene channels with different average grain sizes (see Figure S4 and Figure S5 for *l_G_* = 2.5 and 15 μm, respectively). Following this, to obtain the discrete distribution of the GB number from the continuous gamma PDF, we used the cumulative distribution function of the gamma distribution (i.e., gamma CDF), which is an integral form of the gamma PDF as follows:

(4) $$\begin{array}{c} GamCDF\left(n\right)=\int_{0}^{n}GamPDF\left(x\right)dx\\ \;\;\;\;\;\;\;\;\;\;\;\;\;\;\;\;\;\;\;=\int_{0}^{n}\frac{1}{0.33^{\frac{3.85L_{ch}}{l_{G}}}\int_{0}^{\infty }u^{\left(\frac{3.85L_{ch}}{l_{G}}-1\right)}e^{-u}du}x^{\left(\frac{3.85L_{ch}}{l_{G}}-1\right)}e^{-\frac{x}{0.33}}dx \end{array}$$

where *n* is the number of GBs. The proportion distribution of the GB number can be estimated from *GamCDF* (*n* + 0.5) − *GamCDF* (*n* − 0.5) with an accuracy greater than 98% for all channel lengths, as shown in the red symbols of Figure 2 and Figure S3.

**Figure 3:**
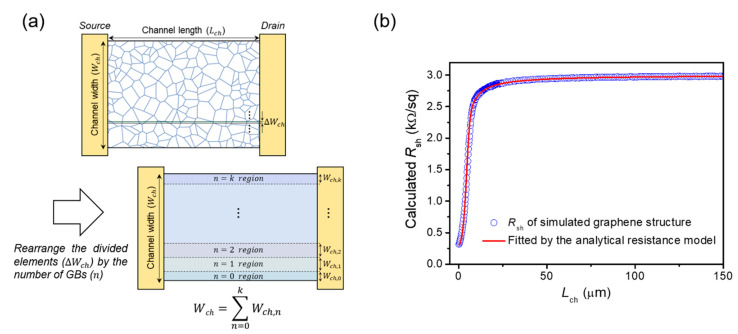
GB distribution-based analytical resistance model for the simultaneous extraction of the grain parameters (the average grain size, grain sheet resistance, GB resistivity) of polycrystalline graphene. (**a**) Considering the proportion distribution of the GB number estimated from the gamma CDF, the divided width elements (Δ*W_ch_*) can be grouped and rearranged by the number of GBs. Based on the rearranged width elements (*W_ch,n_*), a sheet-resistance model composed of the three-grain parameters can be induced as a function of the channel length. (**b**) The developed analytical resistance model was used to fit the calculated channel sheet resistance as a function of the channel length. The sheet-resistance dependence on the channel length can be estimated with a high fitting accuracy greater than 99.98% by adjusting three fit (i.e., grain) parameters, from which the three-grain parameters can be extracted from the analytical resistance model fitted to the *R_sh_*–*L_ch_* curve.

Because the proportion distribution of the GB number within the channel region can now be accurately estimated without counting the number of GBs, we can develop an analytical resistance model that is more generalized for explaining the dependence between the sheet resistance and channel length. For this purpose, the divided narrow width elements (Δ*W_ch_*) were grouped and rearranged by the number of GBs considering its proportion within the channel region estimated from the gamma CDF (Figure 3a). The rearranged width element by the number of GBs (*W_ch,n_*) can be expressed as follows:

(5) $$W_{ch,n}=W_{ch}\times \left\{GamCDF\left(n+0.5\right)-GamCDF\left(n-0.5\right)\right\},\;\;W_{ch}=\overset{k}{\underset{n=0}{\sum }}W_{ch,n}$$

where *k* is the maximum number of GBs existing within the divided width elements. Based on the rearranged width elements, Equation (2) can then be generalized as:

(6) $$\frac{1}{R_{ch}\left(L_{ch}\right)}=\overset{k}{\underset{n=0}{\sum }}\frac{W_{ch,n}}{R_{sh}^{G}L_{ch}+\rho_{GB}n}$$

Following that, the analytical model for the channel sheet resistance, composed of the three-grain parameters (*l_G_*, $R_{sh}^{G}$, and *ρ_GB_*), can be finally induced as a function of the channel length:

(7) $$\begin{array}{c} R_{sh}\left(L_{ch}\right)=R_{ch}\left(L_{ch}\right)\times \frac{W_{ch}}{L_{ch}}\\ ={\left\{\overset{k}{\underset{n=0}{\sum }}\frac{\left(\int_{n-0.5}^{n+0.5}\frac{1}{0.33^{\frac{3.85L_{ch}}{l_{G}}}\int_{0}^{\infty }u^{\left(\frac{3.85L_{ch}}{l_{G}}-1\right)}e^{-u}du}x^{\left(\frac{3.85L_{ch}}{l_{G}}-1\right)}e^{-\frac{x}{0.33}}dx\right)L_{ch}}{R_{sh}^{G}L_{ch}+\rho_{GB}n}\right\}}^{-1} \end{array}$$

Using the developed analytical resistance model as a fitting function, we estimated the dependence of the channel sheet resistance on the channel length observed in the theoretical calculation result, Figure 1c, by adjusting the three unknown fit parameters (i.e., *l_G_*, $R_{sh}^{G}$, and *ρ_GB_*). As a result, the analytical model was fitted with the calculation result well, with a fitting accuracy greater than 99.98% (Figure 3b). Note that any predetermined grain-parameter information is not required in the fitting process. The important aspect of this result is that the three-grain parameters can be extracted from the analytical resistance model that is fitted to the *R_sh_*–*L_ch_* curve. The three-parameter values provided for the theoretical sheet-resistance calculation (Figure 1) and those extracted from the best-fitted analytical model are summarized in Table 1. The result shows that the average grain size, the sheet resistance of single-crystalline grains, and the resistivity of GBs, can be extracted simultaneously with a high accuracy (>99%). Likewise, in the theoretical sheet-resistance results simulated with different average grain sizes or different grain sheet resistance and GB resistivity values, it was also confirmed that the three grain parameters can be extracted with a high accuracy using the analytical model (see Figure S1 and Figure S2). These results indicate that the proposed method can simultaneously extract the average grain size, grain sheet resistance, and GB resistivity of polycrystalline graphene from the electrical characteristics of graphene devices without using any predetermined grain parameter values, and furthermore, it can be applied to various polycrystalline graphene layers with different grain sizes, as well as the electrical properties of GBs and single-crystalline grains.

**Figure 4:**
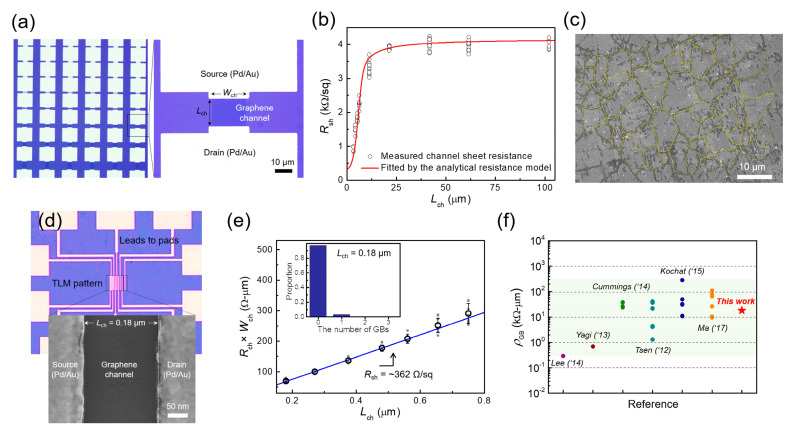
Experimental verification of the GB distribution-based analytical resistance model and parameter extraction method. (**a**) OM images of fabricated TLM patterns comprising the CVD-graphene FETs with varying channel lengths (*L_ch_* of 2–100 μm). (**b**) Measured channel sheet resistance as a function of channel length and fitting result using the analytical resistance model. The three-grain parameters extracted from the fitted model are ~5.95 μm (for the average grain size), ~321 Ω/sq (for the average grain sheet resistance), and ~18.16 kΩ·μm (for the average GB resistivity). (**c**) The representative SEM image of CVD graphene grown on a Cu foil, with UV/ozone-treated GBs highlighted in yellow. The average grain size estimated from 376 grains is 5.88 ± 1.5 μm. (**d**) OM and SEM image of a fabricated TLM pattern comprising the graphene FETs with sub-micrometer channel lengths (*L_ch_* of 0.18–0.75 μm). (**e**) The measured width-normalized channel resistance as a function of the channel length, in which the linear slope indicates the sheet resistance of single-crystalline grains due to the extremely low probability of the presence of GBs within the short-channel regions. Inset: the histogram distribution of the number of GBs when the channel length is significantly smaller than the average grain size (*L_ch_* = 0.18 μm and *l_G_* = 5 μm). (**f**) Summary of experimental results for the GB resistivity reported in the literature [4,5,6,25,26,27,28], where all represented resistivity values were extracted at the charge neutrality point. This summary plot shows that the GB resistivity extracted in this study falls within the range of the reported resistivity values.

To verify whether the proposed parameter extraction method is practical, we fabricated TLM patterns on CVD graphene and extracted three-grain parameters by characterizing the dependence of the channel sheet resistance on the channel length using the analytical resistance model. For this purpose, we synthesized monolayer graphene through CVD and transferred it onto thermally oxidized Si substrates (90-nm-thick SiO_2_) for the device fabrication. The material and layer quality of as-transferred CVD graphene was then evaluated using Raman spectroscopy measurements, confirming the defect-free monolayer graphene with an intensity ratio of 2D to G peaks of ~2.8 (see Figure S6). To characterize the dependence between the channel sheet resistance and the channel length, the back-gated graphene FETs with channel lengths of 2–100 μm and a channel width of 20 μm were fabricated on transferred CVD graphene (Figure 4a) and the total device resistance (at the charge neutrality point) of the graphene FETs was then measured as a function of the channel length. To obtain the channel resistance, the source/drain contact resistance (*R_C_*) was separated from the measured total device resistance (*R_tot_*) by using the TLM method [24]—i.e., *R_ch_* = *R_tot_* − 2*R_C_* (Figure S7). Thereafter, the channel sheet resistance was calculated from *R_ch_* × *W_ch_*/*L_ch_*. The dots in Figure 4b show the channel sheet resistance as a function of the channel length, obtained from the measurement results of 5–15 FETs per channel length using the three identical graphene samples grown by the same CVD run. Note that the dependence between *R_sh_*–*L_ch_* is similar to that observed in the theoretical calculation result (Figure 1c). From such dependence, the three-grain parameters were extracted simultaneously by the analytical resistance model fitted to the *R_sh_*–*L_ch_* curve as shown by the red line in Figure 4b. The extracted average grain size, the sheet resistance of single-crystalline grains, and the resistivity of GBs were determined to be ~5.95 μm, ~321 Ω/sq, and ~18.16 kΩ-μm, respectively. The detailed procedure and flow chart for extracting the three grain parameters from the electrical measurement results using the analytical resistance model are summarized in Figure S7 and Figure S8, respectively.

To confirm whether the extracted three-parameter values are rational, we compared the extracted values with those evaluated using conventional methods and those reported in the literature. First, the GB visualization technique based on UV/ozone treatment was used to estimate the average grain size of CVD graphene. Figure 4c shows a representative SEM image of CVD graphene grown on a Cu foil after the UV/ozone treatment. Note that the UV/ozone-treated GBs were highlighted in yellow to make them more visible (see Figure S9 for the original image). We evaluated the sizes of 376 grains observed in multiple SEM images (Figure S9), from which the average grain size was estimated to be 5.88 ± 1.5 μm. This value agrees well with the extracted average grain size from the fitted analytical resistance model (~5.95 μm). The sheet resistance of single-crystalline grains was estimated by characterizing TLM patterns composed of short-channel graphene FETs with *L*_ch_ of 0.18–0.75 μm (Figure 4d). Because the probability of the GBs existing in these channel lengths is significantly low, the channel resistance increased in direct proportion to the channel length, just like in a single-crystalline material. Thus, the sheet resistance of single-crystalline grains could be estimated using the conventional TLM method [24]—i.e., from the slope of the measured width-normalized channel resistance as a function of the channel length (Figure 4e). The estimated grain sheet resistance from this approach was determined as 362 Ω/sq, which is comparable to the average $R_{sh}^{G}$ extracted from the fitted analytical model (~321 Ω/sq). The slight difference between the two values may be due to the influence of one GB that remains within the channel region, even as the channel length decreases, as shown in the inset of Figure 4e. Accordingly, the grain sheet resistance evaluated using the conventional TLM method can be slightly overestimated because of the carrier scattering at the GB. The extracted GB resistivity from the fitted analytical resistance model (~18.16 kΩ-μm) was verified by comparing the value with those reported in the literature [4,5,6,25,26,27,28]. A summary of experimental results for the GB resistivity of CVD graphene that has been reported in the literature is shown in Figure 4f. Note that all resistivity values shown in the summary plot were extracted at the charge neutrality point. The average GB resistivity value in this study is in the range of those reported in the literature to date and is similar to their average value. Consequently, these validation results support that the extracted three-grain parameters are within the rational range. Therefore, we can conclude that the proposed electrical characterization method can extract the average grain size, single-crystalline grain sheet resistance, and GB resistivity simultaneously using the GB distribution-based analytical resistance model. This method of simultaneous extraction of the grain-related parameters from the dependence between *R_sh_*–*L_ch_* obtained from simple TLM measurements will provide a convenient way for the electrical characterization of CVD graphene and its efficient device applications. Furthermore, it is expected that the proposed method can be extended to various 2D materials with polycrystalline structures.

## 4. Conclusions

In summary, we demonstrated an electrical characterization method for extracting the average grain size, grain sheet resistance, and GB resistivity of monolayer CVD graphene simultaneously. We developed an analytical resistance model to explain the relationship between the electrical properties of polycrystalline graphene and the channel dimension by precisely estimating the proportion distribution of the number of GBs within graphene channels. With the developed analytical resistance model, we showed that the three-grain parameters can be extracted simultaneously from the dependence of the graphene sheet resistance on the channel dimension with an accuracy greater than 99%. The proposed parameter extraction method using the GB distribution-based analytical resistance model was experimentally verified by characterizing TLM patterns fabricated on monolayer CVD graphene. The result showed that the average grain size, grain sheet resistance, and GB resistivity of CVD graphene can be extracted simultaneously with high accuracy from the analytical resistance model fitted to the measured *R_sh_*–*L*_ch_ curve. We believe that this method will be a useful tool for the electrical characterization of CVD graphene and other polycrystalline 2D materials.

## Figures and Tables

**Table 1:** Comparison of three-grain parameters given for the theoretical calculation with those extracted by the developed analytical resistance model.

	*l_G_* (μm)	$R_{sh}^{G}$ (Ω/sq)	*ρ_GB_* (kΩ-μm)
Given parameter	5.0	300	10.6
Extracted parameter	5.02	299.7	10.59

## Data Availability

Data supporting the results are presented in the article and Supplementary Materials in the form of graphs, tables, and schematic diagrams. Raw and/or additional data are available on request from the corresponding author.

## Supplementary Materials

The following are available online at https://www.mdpi.com/article/10.3390/nano12020206/s1, MATLAB ^®^ algorithm/code file used for the grain-parameter extraction, Figure S1: Theoretically-calculated *R_ch_*–*L_ch_* and *R_sh_*–*L_ch_* results for polycrystalline graphene with different average grain sizes and the grain-parameter extraction results using the analytical resistance model, Figure S2: Theoretically-calculated *R_ch_*–*L*_ch_ and *R_sh_*–*L_ch_* result using different *W_ch_*, $R_{sh}^{G}$, and *ρ_GB_* values and the grain-parameter extraction results using the analytical resistance model, Figure S3: Average histogram distributions of the proportion of the number of GBs within the channel region at various channel lengths, Figure S4: Average histogram distributions of the proportion of the number of GBs within the channel region for polycrystalline graphene with an average grain size of 2.5 μm, Figure S5: Average histogram distributions of the proportion of the number of GBs within the channel region for polycrystalline graphene with an average grain size of 15 μm, Figure S6: Raman spectrum of the graphene layer grown by CVD, Figure S7: A procedure for measuring the graphene sheet resistance as a function of the channel length and extracting the three grain parameters using the analytical resistance model, Figure S8: A flow chart explaining the algorithm for the process of extracting grain parameters using the analytical resistance model, Figure S9: Top-view SEM images of UV/ozone-treated CVD graphene, used for estimation of the average grain size.
