# Supplementary material for: Simultaneous Extraction of the Grain Size, Single-Crystalline Grain Sheet Resistance, and Grain Boundary Resistivity of Polycrystalline Monolayer Graphene
Source: Nanomaterials (Basel). 2022 Jan 9;12(2):206. doi: 10.3390/nano12020206 (PMC8781743; doi:10.3390/nano12020206)
Supplement: Supplementary file 1 [file nanomaterials-12-00206-s001.zip › nanomaterials-1510991-supplementary.pdf]

nanomaterials

MDPI

Supplementary Material:

Simultaneous Extraction of the Grain Size, Single-Crystalline Grain Sheet Resistance, and Grain Boundary Resistivity of Polycrystalline Monolayer Graphene

Honghwi Park [1], Junyeong Lee [1], Chang-Ju Lee [1], Jaewoon Kang [1], Jiyeong Yun [1], Hyowoong Noh [1], Minsu Park [1], Jonghyung Lee [1], Youngjin Park [1], Jonghoo Park [1], Muhan Choi [1,2], Sunghwan Lee [3] and Hongsik Park [1,2,*]

[1] *School of Electronic and Electrical Engineering, Kyungpook National University, Daegu 41566, South Korea*

[2] *School of Electronics Engineering, Kyungpook National University, Daegu 41566, South Korea*

[3] *School of Engineering Technology, Purdue University, West Lafayette, IN 47907, USA*

* Correspondence: hpark@ee.knu.ac.kr (H.P.)

This PDF file includes:

Figures S1 to S9

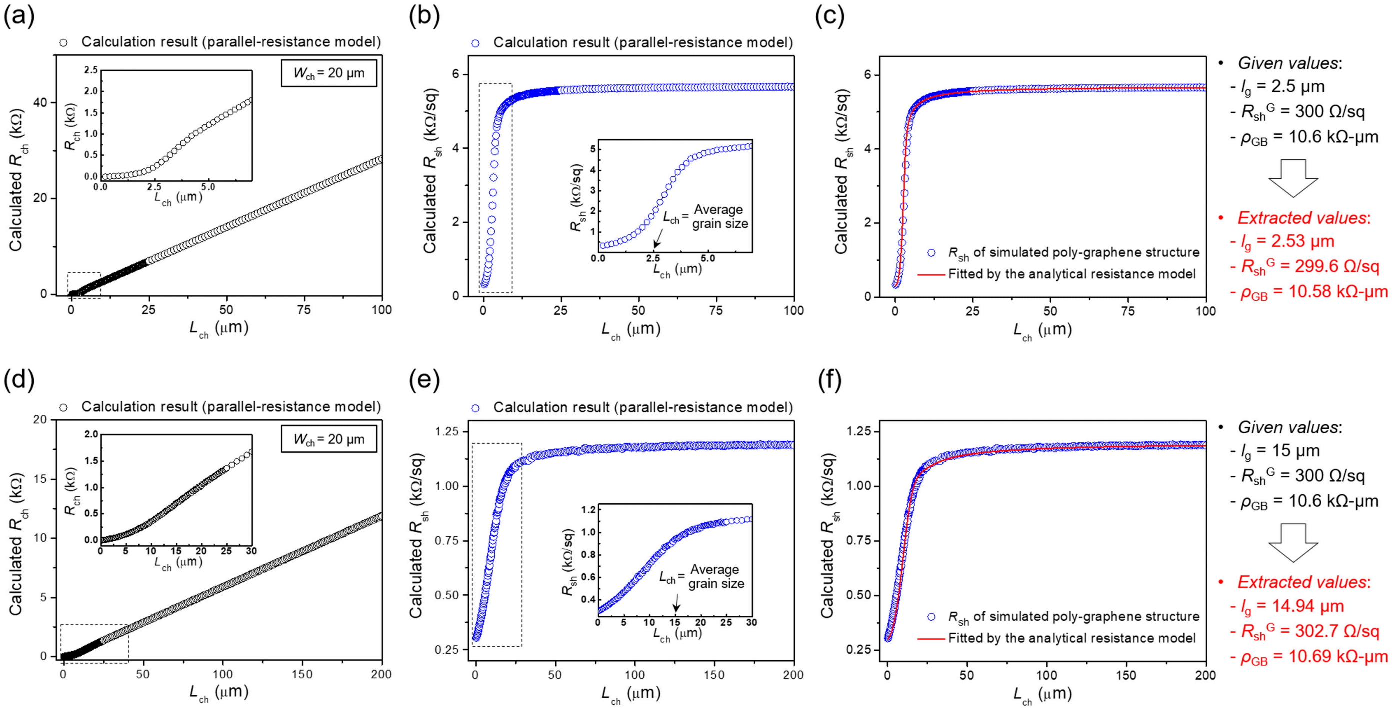

Figure S1. (**a**) The theoretically-calculated R_{ch}–L_{ch} and (**b**) R_{sh}–L_{ch} result for polycrystalline graphene simulated with an average grain size of 2.5 μm, and (**c**) the grain-parameter extraction using the analytical resistance model and comparison of three-grain parameters given for the theoretical calculation with those extracted by the fitted analytical model. (**d**) The theoretically-calculated R_{ch}–L_{ch} and (**e**) R_{sh}–L_{ch} result for polycrystalline graphene simulated with an average grain size of 15 μm, and (**f**) the grain-parameter extraction using the analytical resistance model and comparison of three-grain parameters given for the theoretical calculation with those extracted by the fitted analytical model. The channel width used for the calculation processes was 20 μm. For both cases with the different grain sizes, the three-parameter values provided for the theoretical computation and those extracted from the best-fitted analytical model agreed well with a difference less than 1%. This result implies that the proposed characterization method using the analytical model can be applied to extract the grain parameters of various polycrystalline graphene layers with different average grain sizes.

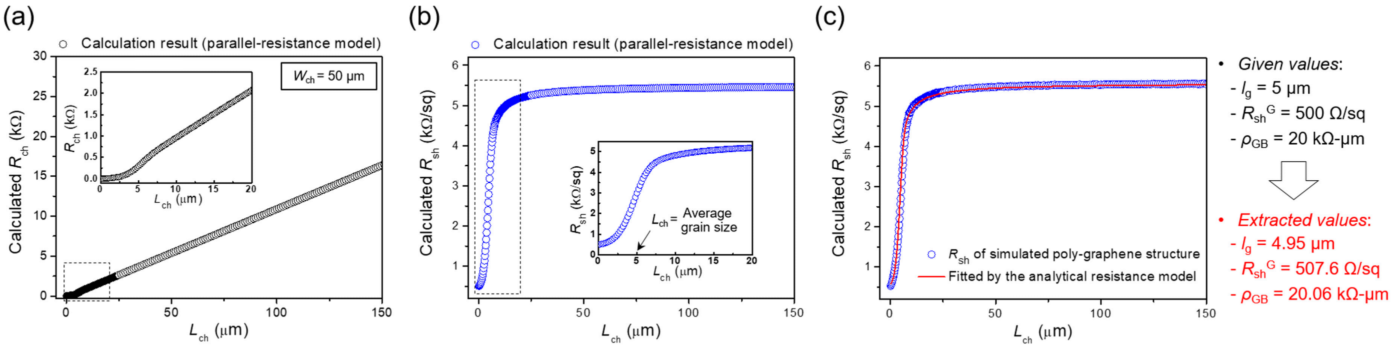

Figure S2. (**a**) The theoretical R_{ch}–L_{ch} and (**b**) R_{sh}–L_{ch} result calculated using different W_{ch} (50 μm), R_{sh}^{G} (500 Ω/sq), and ρ_{GB} (20 kΩ-μm) values. These R_{sh}^{G} and ρ_{GB} values were also selected within the range of those of CVD-grown polycrystalline graphene reported in the literature. (**c**) The grain-parameter extraction using the analytical resistance model and comparison of three-grain parameters given for the theoretical calculation with those extracted by the fitted analytical model. The three grain parameters extracted from the best-fitted analytical model are in good agreement with those values provided for the theoretical calculation (with a difference less than 1%). This result implies that the proposed analytical model can also be applied to extract the grain parameters of various polycrystalline graphene layers with different electrical properties of grains and GBs.

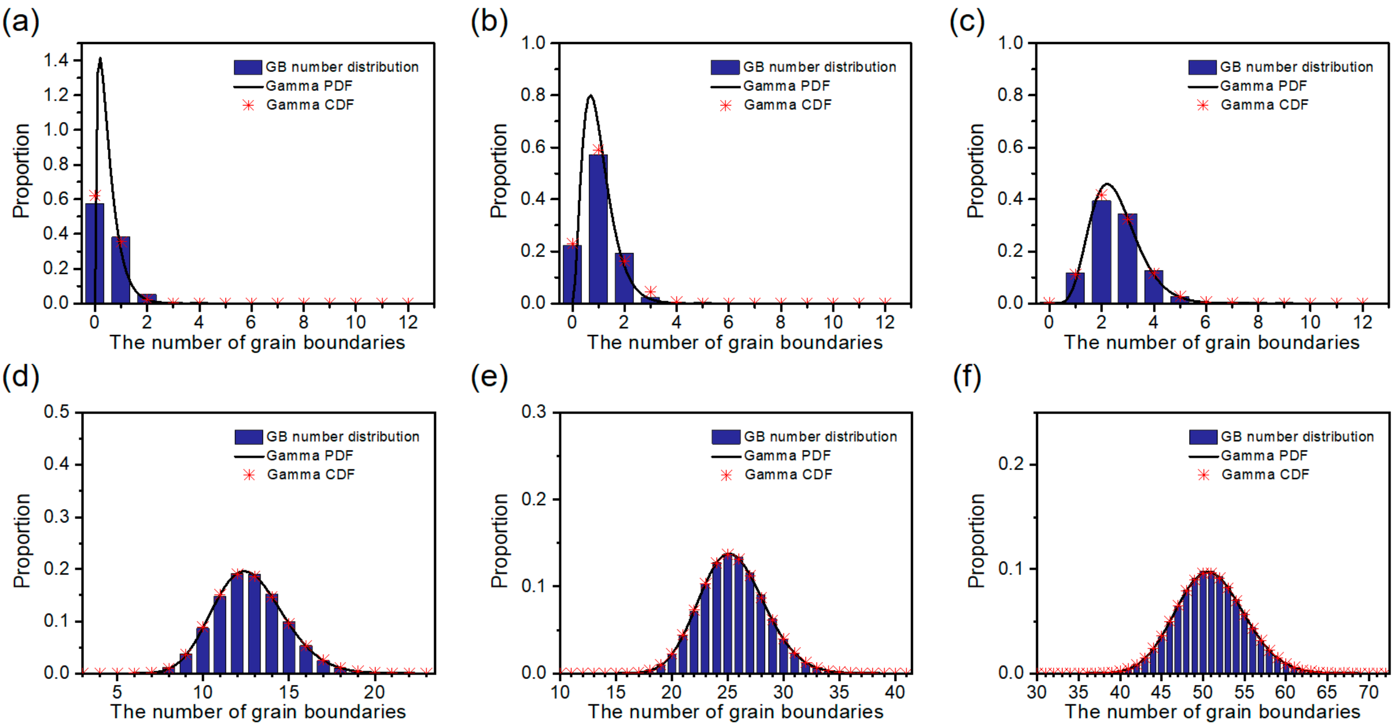

Figure S3. Average histogram distributions of the proportion of the number of GBs within the channel region at (**a**) L_{ch} = 2 µm, (**b**) L_{ch} = 4 µm, (**c**) L_{ch} = 10 µm, (**d**) L_{ch} = 50 µm, (**e**) L_{ch} = 100 µm, and (**f**) L_{ch} = 200 µm. For all the channel lengths, the envelope of the proportion distribution of the number of GBs follows the continuous gamma PDF, and the discrete proportion distributions of the GB number can be estimated accurately based on the gamma CDF [*i.e.*, $GamCDF(n+0.5) - GamCDF(n-0.5)$].

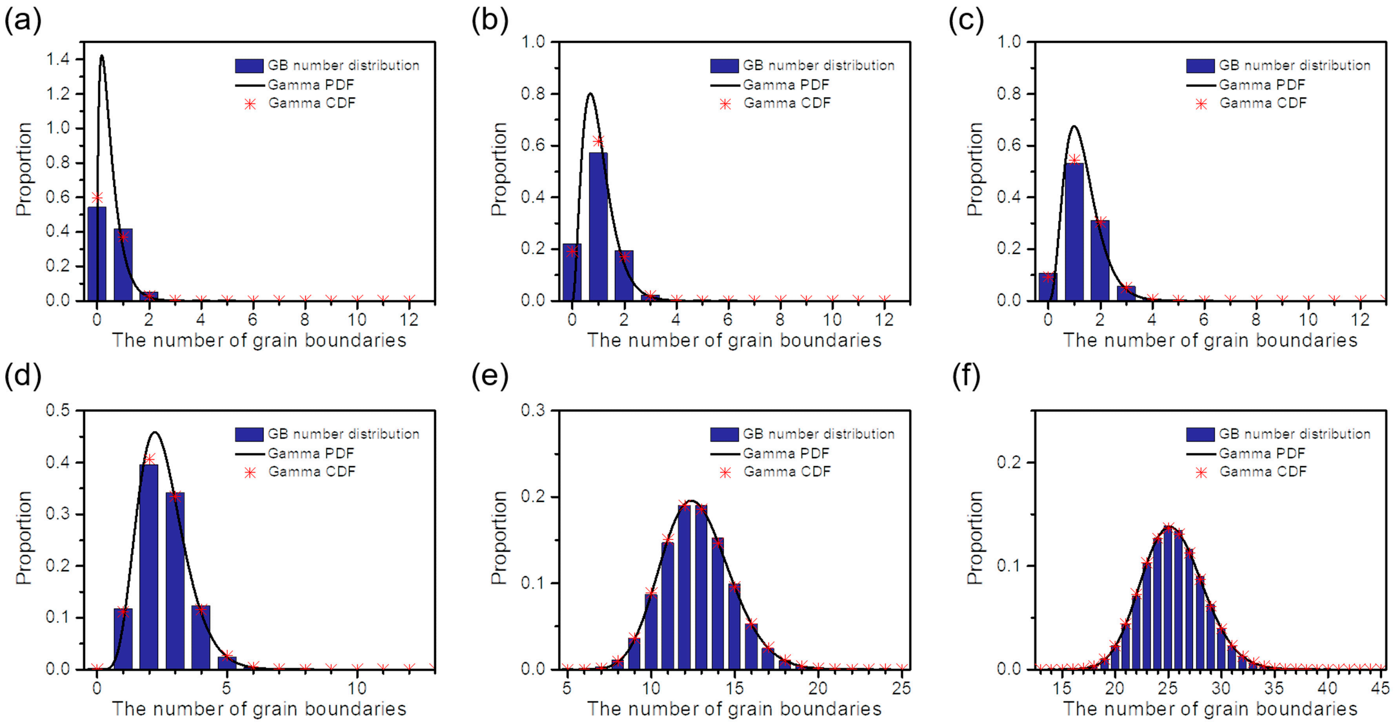

Figure S4. For polycrystalline graphene with an average grain size of 2.5 μm, average histogram distributions of the proportion of the number of GBs within the channel region at (**a**) L_{ch} = 1 μm, (**b**) L_{ch} = 2 μm, (**c**) L_{ch} = 2.5 μm, (**d**) L_{ch} = 5 μm, (**e**) L_{ch} = 25 μm, and (**f**) L_{ch} = 50 μm. For all the channel lengths, the envelope of the proportion distribution of the number of GBs follows the continuous gamma PDF with $\alpha = 3.85 \times L_{ch}/l_G$ and $\beta = 0.33$. The discrete proportion distributions of the GB number can be estimated accurately based on the gamma CDF [*i.e.*, $GamCDF(n+0.5) - GamCDF(n-0.5)$].

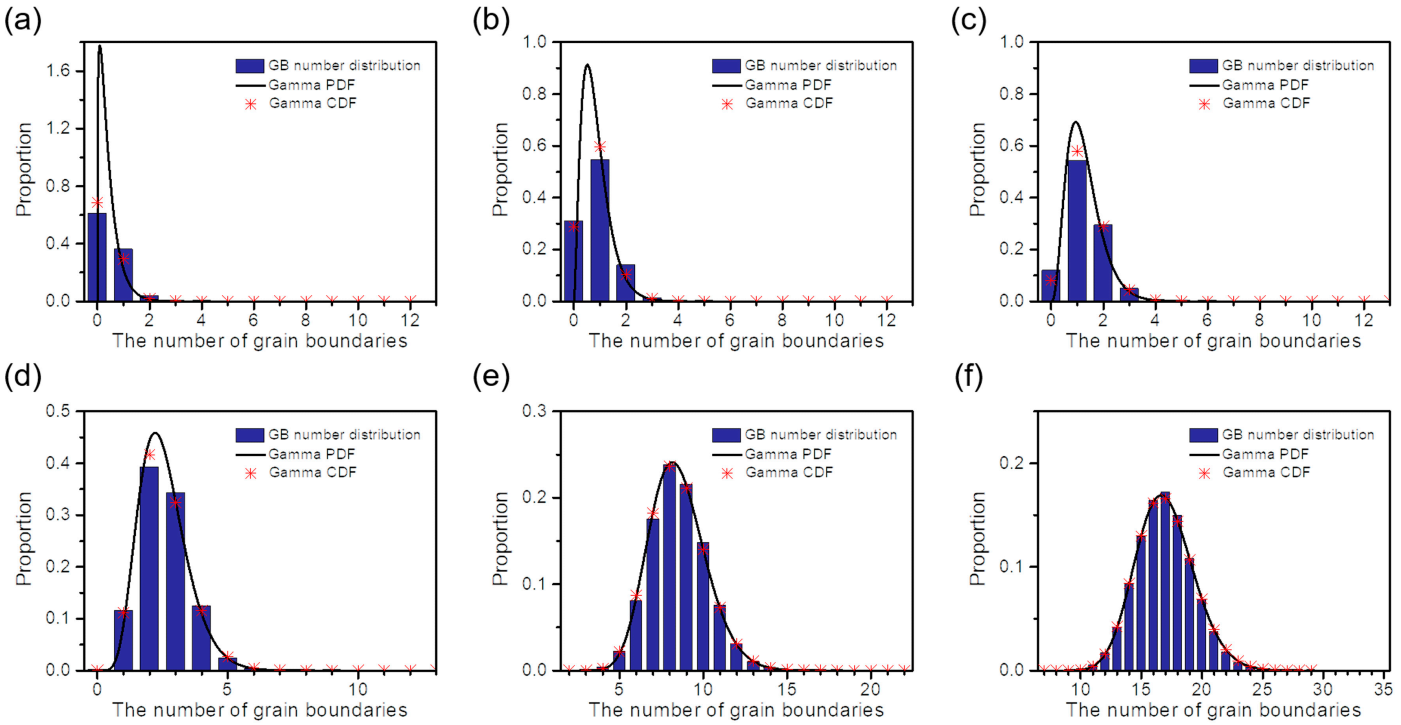

Figure S5. For polycrystalline graphene with an average grain size of 15 µm, average histogram distributions of the proportion of the number of GBs within the channel region at (**a**) L_{ch} = 5 µm, (**b**) L_{ch} = 10 µm, (**c**) L_{ch} = 15 µm, (**d**) L_{ch} = 30 µm, (**e**) L_{ch} = 100 µm, and (**f**) L_{ch} = 200 µm. For all the channel lengths, the envelope of the proportion distribution of the number of GBs follows the continuous gamma PDF with $\alpha = 3.85 \times L_{ch}/l_G$ and $\beta = 0.33$. The discrete proportion distributions of the GB number can be estimated accurately based on the gamma CDF [*i.e.*, $GamCDF(n+0.5) - GamCDF(n-0.5)$].

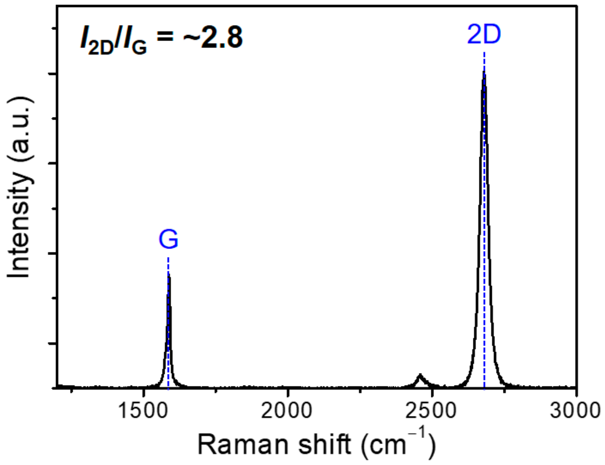

Figure S6. Raman spectrum of the graphene layer grown by CVD. The intensity ratio of 2D to G peaks is evaluated to be approximately 2.8, and no noticeable D peak (corresponding to a crystal defect) is observed in the spectrum. This indicates that CVD graphene used for the FET fabrication is a monolayer with minimized defects.

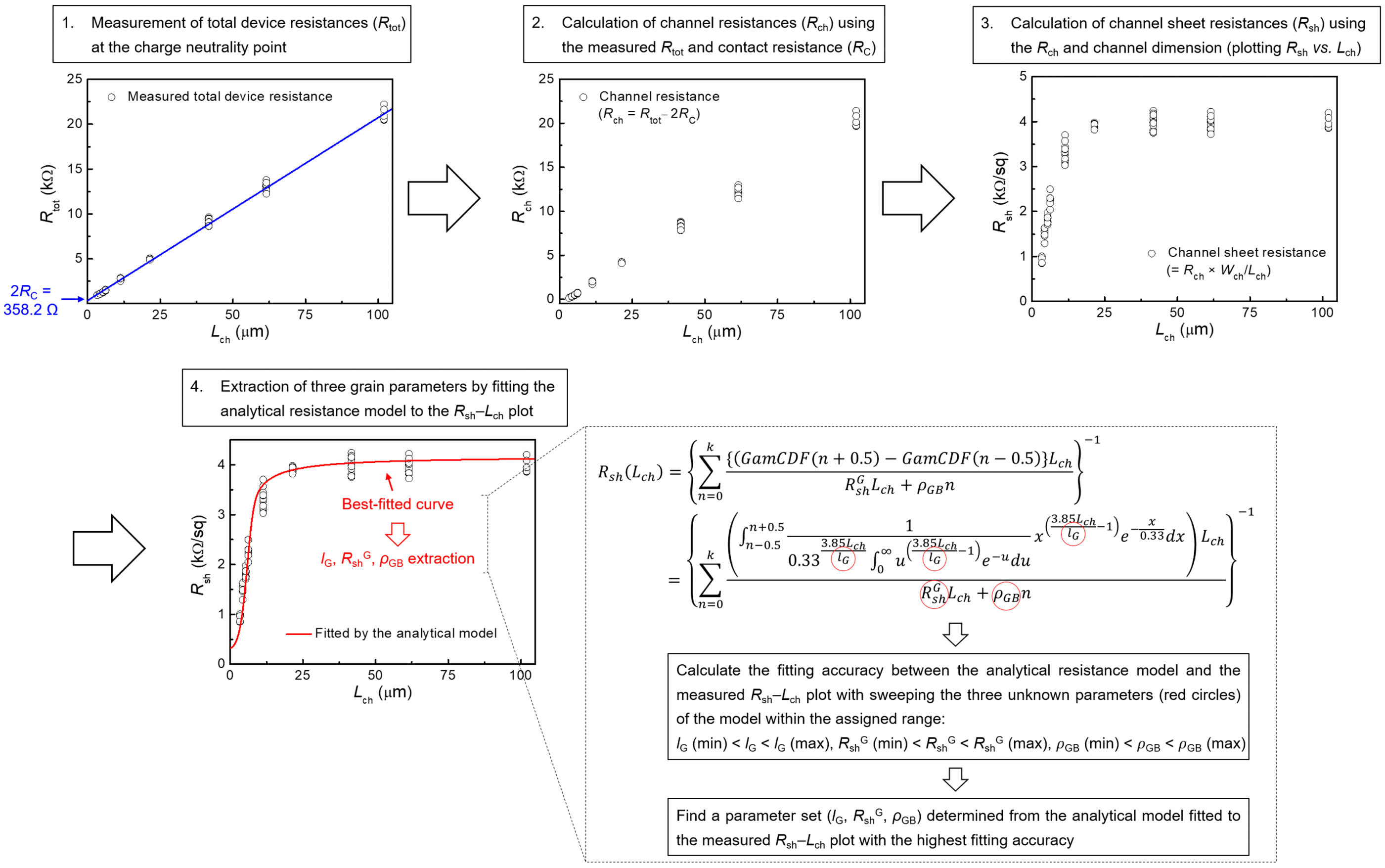

$$R_{sh}(L_{ch}) = \left\{\sum_{n=0}^{k} \frac{\{(GamCDF(n+0.5) - GamCDF(n-0.5)\}L_{ch}}{R_{sh}^G L_{ch} + \rho_{GB} n}\right\}^{-1}$$

$$= \left\{\sum_{n=0}^{k} \frac{\left(\int_{n-0.5}^{n+0.5} \frac{1}{0.33^{\frac{3.85L_{ch}}{l_G}} \int_0^{\infty} u^{\left(\frac{3.85L_{ch}}{l_G}-1\right)} e^{-u} du} x^{\left(\frac{3.85L_{ch}}{l_G}-1\right)} e^{-\frac{x}{0.33}} dx\right) L_{ch}}{R_{sh}^G L_{ch} + \rho_{GB} n}\right\}^{-1}$$

Figure S7. A procedure for measuring the graphene sheet resistance as a function of the channel length and extracting the average grain size, grain sheet resistance, and GB resistivity by fitting the analytical resistance model to the measured R_{sh}–L_{ch} plot.

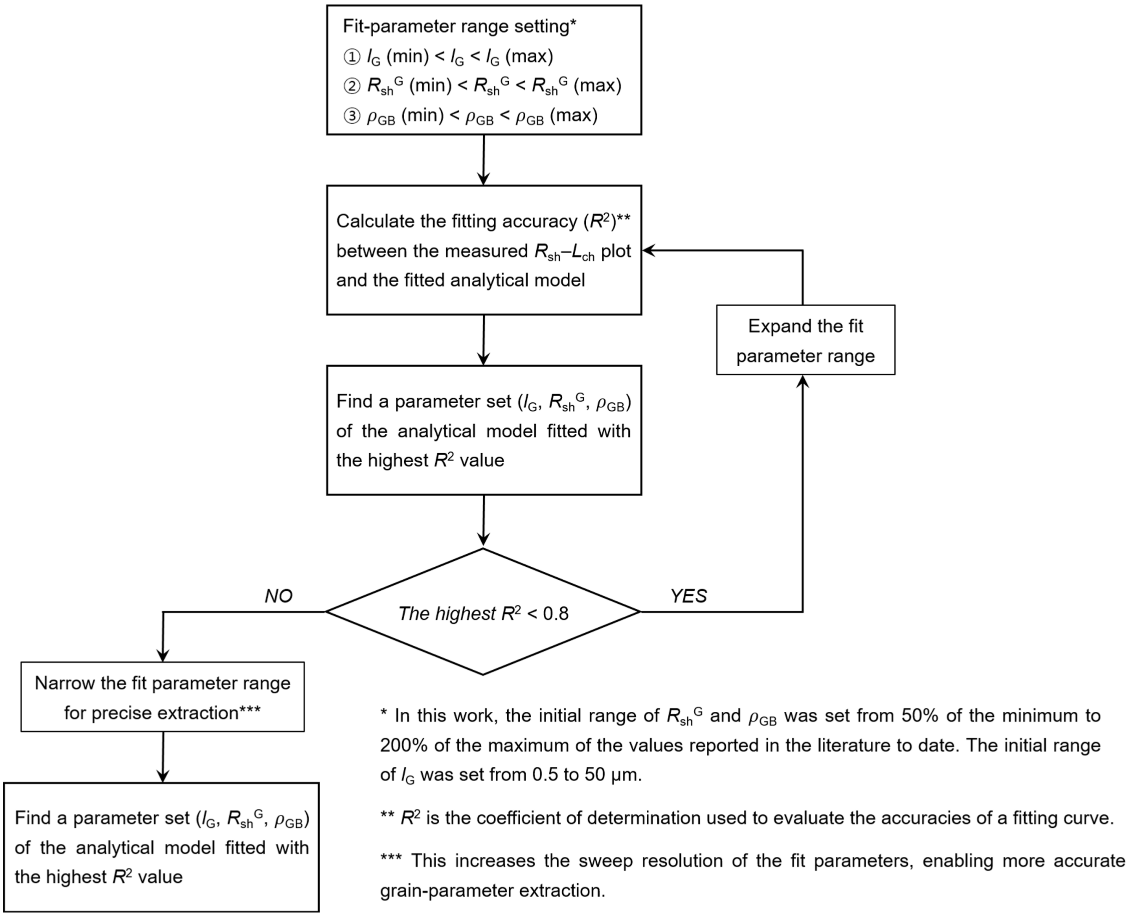

Figure S8. A flow chart explaining the algorithm for the process of extracting grain parameters using the analytical resistance model.

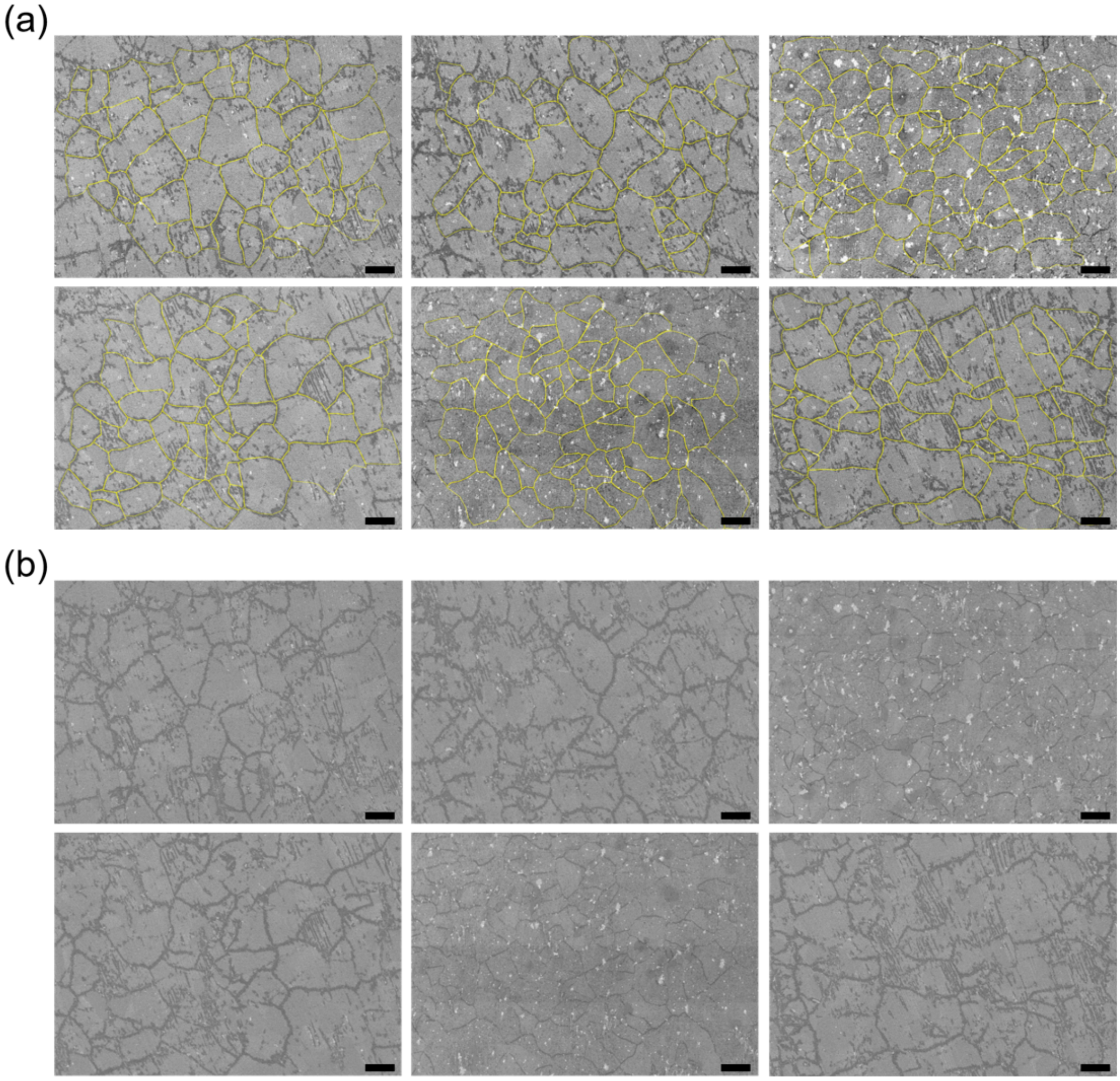

Figure S9. Top-view SEM images of UV/ozone-treated CVD graphene, used for estimation of the average grain size: (**a**) The images with UV/ozone-treated GBs highlighted in yellow and (**b**) their original images. The average grain size estimated from 376 grains is 5.88 ± 1.5 μm. Scale bars in all SEM images are 5 μm.
